# Telerehabilitation is Effective to Recover Functionality and Increase Skeletal Muscle Mass Index in COVID-19 Survivors

**DOI:** 10.5195/ijt.2021.6415

**Published:** 2021-12-16

**Authors:** Jorge Cancino-López, Patricio Zarricueta Vergara, Bárbara Leyton Dinamarca, Pedro Figueroa Contreras, Luis Miño Cárcamo, Nicolás Cartagena Ibarra, Johana Soto-Sánchez

**Affiliations:** 1 Exercise Physiology and Metabolism Laboratory, Medicine Faculty, Finis Terrae University, Santiago, Chile; 2 Sports Corporation of Florida, Chile; 3 Institute Of Nutrition And Food Technology (INTA), University of Chile, Santiago, Chile; 4 Department of Physical Activity, Faculty of Sciences of Physical Activity and Sports, Research Laboratory in Physical Activity and Sport. University of Playa Ancha, Valparaiso, Chile

**Keywords:** Barthel index, COVID-19, Hand grip strength, Skeletal muscle mass, Physical therapy, Telerehabilitation

## Abstract

**Objective:**

The purpose of this study was to evaluate the effects of a telerehabilitation program for COVID-19 survivors on their functionality, aerobic capacity, upper-lower body strength and skeletal muscle mass index.

**Methods:**

Fifty patients (22 M); age 54.1±15.4 who became ill with COVID-19 during 2020 completed a 24-session telerehabilitation program. The following measures were taken: Barthel's index, two minutes step test (2MST), elbow flexion one-repetition maximal (1RM), short physical performance battery (SPPB), hand grip strength, 30-second chair stand, skeletal muscle index (SMI), body fat percentage, resting pulse, arterial blood pressure, and pulse oximetry.

**Results:**

There was a significant increase in the Barthel index (p≤0.0001), 2MST (p≤0.0001), 1RM elbow flexion (p≤0.0001), SPPB (p≤0.0001), hand grip strength (p≤0.0001), 30-second chair stand (p≤0.000l), and SMI (p≤0.0001).

**Conclusion:**

A 24 session in-home telerehabilitation program promoted the recovery of physical independence and increases in skeletal muscle mass index and physical fitness.

One of the challenges faced by persons who become infected by the SARS-CoV-2 virus, develop severe symptoms, and survive, is the restoration of their diminished functional capacity (Rooney et al., 2020). It has been reported that more than 40% of surviving COVID-19 patients worsen their quality of life with persistence of symptoms for more than 60 days (Carfì et al., 2020). The time spent in bed as a result of hospitalization, along with medications and nutritional conditions linked to patent sedation, will cause a significant reduction in body weight, mainly affecting skeletal muscle mass and influencing the muscle strength development (Di Filippo et al., 2021; Wischmeyer et al., 2017). The reduction in physical independence evaluated according to the Barthel index for survivors of COVID-19 is around 70% (Belli et al., 2020). Survivors of the disease who are younger and who have undergone prolonged hospitalizations often find their physical independence negatively affected; this directly impacts their return to activities of daily life and work and diminishes their quality of life (Jacobs et al., 2020). Also, some intensive care survivors may be affected by post intensive care syndrome with persistent cognitive and physical limitations.

Due to the necessity for social distancing during the pandemic, telecommunication has proved to be valuable in supporting work, life, and health care. Concurrently, telerehabilitation has enabled the provision of live audio and video patient care thereby optimizing remote access and safe care (Prvu Bettger & Resnik, 2020; Werneke et al., 2021).

The purpose of this study was to document the results of a telerehabilitation program for COVID-19 survivors, specifically: functionality, aerobic capacity, upper and lower body strength, skeletal muscle mass index, and cardiovascular and respiratory variables.

## METHODS

### DESIGN

This study was observational and was conducted between September 2020 and February 2021. Patients were individually treated for a total 24 telerehabilitation sessions, each with a duration of 50-60 minutes. Data were collected from clinical records. Four physiotherapists conducted the sessions remotely by phone video call. Each session provided personalized clinical attention to patients. The physiotherapist maintained constant visual contact and corrected the exercises for proper execution. Participants at the time of the initial evaluation received a training kit consisting of two dumbbells of 2 kg each; one exercise step; a closed blue elastic band; a blue open elastic band; and a 65 cm diameter physio ball. Evaluations were carried out at session 1, session 12, and session 24.

### PARTICIPANTS

Fifty patients (22 M, 28 F), age 54.1 ± 15.4 (range: 24-86), BMI 29.9 ± 4.96 kg/m^2^ (range: 18.9-44.3) who became ill with COVID-19 during 2020 were entered into a telerehabilitation program of physical therapy. The telerehabilitation program's inclusion criteria were three or more of the following: Barthel's index ≥60 points, or value of dyspnea at rest ≤2 (0 to 10 scale), Short Physical Performance Battery (SPPB) score ≥10, or hand strength with Handgrip test ≤60 kg in men and ≤40 kg in women, or indirect measurement of the maximum dynamic force of elbow flexors with a value ≤12 kg in men and ≤10 kg in women, or aerobic capacity test with 2 minute step test (TS2M) in men ≤100 repetitions and in women ≤90 repetitions, or lower body strength with the 30-S chair stand test, where a value ≤25 repetitions in men and ≤20 in women. The telerehabilitation program's exclusion criteria were: cognitive and intellectual disability, previous motor disability, and/or being a woman in a gestation period.

### ETHICAL ASPECTS

The participants signed an informed consent upon their agreement to participate in the study. The research was approved by the ethics committee of the University Finis Terrae N° 12-05-2021.

### INTERVENTION

The intervention consisted of 24 sessions with the patient at home, 2-3 times a week, with a session duration of 50-60 minutes. Four therapists, specialists in clinical exercise physiology and rehabilitation communicated by videotelephony from the health center to the homes of the participants.

The structure of the session was: 10 minutes warm-up, 25 minutes resistance training, 10 minutes aerobic training, and 5 minutes cool-down. Participants received a training kit at the time of the initial evaluation. The intensity of the session was controlled with the Borg's scale of perception of effort (Borg, 1982) and kept at a value of 12 -13 (somewhat hard). A mid-study evaluation was carried out in session 12. The program was considered complete when each subject completed 24 sessions.

### OUTCOME MEASURES

#### FUNCTIONALITY

Patients answered the Barthel index of Activities of Daily Living, which determines the level of dependence to perform 10 activities of daily life (Cid-Ruzafa & Damian-Moreno, 1997).

#### PHYSICAL FITNESS ASSESSMENT

The Short Physical Performance Battery (SPPB) was performed (Rodriguez-Manas et al., 2014). SPPB is comprised of three subtests: balance tests, gait speed test and chair stand test. Values below 10 indicate fragility and high risk of falling.

#### AEROBIC EXERCISE CAPACITY

Aerobic exercise capacity was evaluated via the 2-minute step test, which corresponds to the highest number of motor gestures of the gait in place, in 2 minutes. It is counted when the right leg reaches the predefined height (Rikli & Jones, 2013).

#### HAND STRENGTH

Hand strength was measured with the Handgrip test (Fess, 1992). The seated participant took the dynamometer (Baseline Lite Tm 12-0241, USA) with the dominant hand, and in 90° elbow flexion, pressing as much as possible; the equipment was adjusted in order to allow a comfortable grip. The best of two attempts was recorded.

#### ELBOW FLEXOR MUSCLE STRENGTH

Elbow flexor muscle strength was measured by maximal repetition numbers in flexion and extension of both elbows simultaneously with 2 or 5 kg dumbbell, depending on tolerance. Then Epley's one maximal repetition (1RM) equation was applied and 1RM in kg calculated (Reynolds et al., 2006).

#### LOWER BODY STRENGTH

Lower body strength was measured through the 30-s chair stand test (30-s CST). The maximal number of successful executions in thirty seconds was recorded (Jones et al., 1999).

#### ANTHROPOMETRIC, CARDIOVASCULAR, AND RESPIRATORY VARIABLES

Body weight, fat percentage, and skeletal muscle mass were measured by segmental, multi-frequency, and multipolar bioelectrical impedance (Tanita BC-1500 plus, Japan). Then, SMI was calculated by dividing the appendicular muscle mass sum by square of the height. Waist circumference was measured at the narrowest point visually detected (Norton & Olds, 1995). Blood pressure was measured in the left arm in a sitting position with a digital device (Nissei DS-11, Japan). Both oxygen saturation and pulse rate were measured with a digital oximeter (Oxywatch MD300C11, China), and dyspnea perception at rest was queried with the modified Borg scale (Kendrick et al., 2000)

### DATA ANALYSIS

Descriptive statistics were performed through the median, percentile 25-75 since the variables did not have a normal distribution; this was confirmed through the Shapiro Wilk test. To compare the results between the start of the intervention with 12 sessions, and the start of the intervention with 24 sessions, the Wilcoxon test was performed for paired samples and the effect size was determined by calculating Cliff's Delta (δ) non-parametric test. Cliff's Delta threshold values for small, moderate and large effect was 0.2, 0.5, 0.8 respectively. In addition, for the analysis of the dyspnea variable, the symmetry test was assessed at start of the program and at 24 sessions (changes are expressed as a percentage). A significant difference was considered as a p value ≤0.05. All analyses were performed with the statistical software STATA 16.

## RESULTS

### FUNCTIONALITY AND PHYSICAL FITNESS

Positive changes were observed that indicated that with only 12 sessions (δ=0.794) independence was recovered in all survivors of COVID-19, and was maintained in session 24 (δ=0.849). Regarding the physical condition, positive changes were observed in SPPB at 12 and 24 sessions (δ=0.646 and 8:0.795 respectively). Regarding the 2MST, an increase in the number of repetitions were observed both in session 12 (δ=0.875) and in session 24 (δ=0.875). Increases in absolute handgrip strength were seen at 12 (δ=0.743) and 24 sessions (δ=0.798). The 1RM dumbbell elbow flexion also increased at 12 sessions (δ=0.793) and 24 sessions (δ=0.818). In regard to the lower body muscle strength, there was an increase in repetition number in the 30-s CST at 12 (δ=0.833) and 24 sessions (δ=0.818), (Table 1).

**Table 1:** Modifications on Functionality and Physical Fitness at 12 and 24 Sessions

Variables	Pre intervention		12 sessions		24 sessions		P1		P2	
	n	Mediana (p25-p75)	n	Mediana (p25-p75)	n	Mediana (p25-p75)	p value	Effect size‡	p value	Effect size‡
***Functionality***										
Barthel Index (sc)	27	95(85–95)	26	100 (100–100)	27	100 (100–100)	≤0.0001*	0.794	≤0.0001*	0.849
***Physical fitness***										
Short physical performance Battery (sc)	25	11 (9–12)	24	12 (11.5–12)	25	12 (12–12)	0.0012*	0.646	≤0.0001*	0.795
2MST (rs)	25	57 (45–66)	24	66 (60.5–81)	25	80 (70–89)	<0.0001*	0.875	≤0.0001*	0.875
Borg scale 2MST (6–20)	25	13 (13–15)	24	12 (13–15)	25	15 (11–15)	0.5654	0.124	0.7552	0.069
Handgrip (kg)	27	28 (19–34)	26	28.5(26–38)	27	32 (28–36)	≤0.0001*	0.743	≤0.0001*	0.798
Handgrip (kg)/body weight (kg)	27	0.32 (0.25–0.40)	26	0.36 (0.32–0.44)	27	0.38 (0.34–0.44)	≤0.0001*	0.695	≤0.0001*	0.744
1RM Dumbbell Elbow flexion (kg)	45	4.7 (3.7–5.9)	43	7.5 (5.8–8.5)	45	8.8 (7.2–10)	≤0.0001*	0.793	≤0.0001*	0.818
30-s chair stand (rs)	37	14 (11–16)	35	17 (15–19)	37	20 (17–21)	≤0.0001*	0.833	≤0.0001*	0.867

*Note*. sc: score. rs: repetitions. 2MST: 2-minute step test P1: modifications between the start of the intervention and 12 sessions. P2: modifications between the start of the intervention and 24 sessions.

‡ : Effect size by Cohen's d.

All data was analyzed with pared Wilcoxon test.

### ANTHROPOMETRIC, CARDIOVASCULAR AND RESPIRATORY VARIABLES

Table 2 shows how the surviving patients of COVID-19 maintained or modified the anthropometric and body composition variables. With respect to body weight, positive changes were observed at 12 (δ=0.466) and 24 sessions (δ=0.625) and for BMI an improvement was observed both at 12 sessions (δ=0.474) and at 24 sessions (δ=0.630). Both for the waist circumference, as well as the waist / height ratio, no changes were observed after 24 sessions. An increase in muscle mass was observed both in session 12 (δ=0.555) and in session 24 (δ=0.761). Finally, the SMI showed an increase in session 24 (δ=0.697).

**Table 2:** Modifications on Anthropometrics and Body Composition at 12 and 24 Sessions

Variables	Pre intervention		12 sessions		24 sessions		P1		P2	
	n	Mediana (p25-p75)	n	Mediana (p25-p75)	n	Mediana (p25-p75)	p value	Effect size‡	p value	Effect size‡
Weight (kg)	50	76.1 (64.4–93.4)		78.1 (68.9–91.1)	48	78.9 (71.5–95.2)	0.0009*	0.466	≤0.0001*	0.625
BMI (kg/m^2^)	50	29.71 (26.42–33.02)	48	30.43 (27.26–33.21)	50	30.98 (27.82–33.74)	0.0008*	0.474	≤0.0001*	0.630
Waist + (cm)	50	98 (92–107)	48	97.5(90.5–104)	50	98 (92–106)	0.7654	0.044	0.0500	0.277
Waist/Height index	50	0.61 (0.57–0.65)	48	0.60 (0.56–0.65)	50	0.61 (0.56–0.66)	0.8486	0.028	0.0539	0.272
Body Fat (%)	50	38.15 (31.5–43.5)	48	37.4 (31.35–41.35)	50	36.95 (33.1–39.5)	0.1178	0.227	0.0402*	0.289
Muscle mass (kg)	50	45.95 (38.8–52.7)	48	47.3 (39.4–56.75)	50	48.7(42–57.2)	≤0.0001*	0.555	≤0.0001*	0.761
SMI (kg/m^2^)	41	7.84 (7.16–8.37)	39	7.9 (7.3–8.7)	41	8.2 (7.8–8.8)	0.0704	0.291	≤0.0001*	0.697

*Note*. BMI: Body mass index. SMI= Skeletal muscle mass index. P1: modifications between the start of the intervention and 12 sessions. P2: modifications between the start of the intervention and 24 sessions.

‡ : Effect size by Cohen's d.

All data was analyzed with pared Wilcoxon test.

Regarding the cardiovascular variables (Table 3), a progressive increase in pulse rate in session 24 (δ=0.549) was observed at the end of physical work. With regard to the systolic blood pressure at the beginning of each session, a decrease was observed as the program progressed [12 sessions (δ=0.409) and 24 sessions (δ=0.425)]. For the double product, there was a progressive decrease at 12 (δ=0.456) and 24 sessions (δ=0.330). In contrast, the double product at the post session 24 increased with respect to the baseline values (δ=0.596).

**Table 3:** Modifications Cardiovascular and Respiratory Parameters at 12 and 24 Sessions

Variables	Pre intervention		12 sessions		24 sessions		P1		P2	
	n	Mediana (p25-p75)	n	Mediana (p25-p75)	n	Mediana (p25-p75)	p value	Effect size‡	p value	Effect size‡
***Cardiovascular***										
PR PS (beats/min)	50	82.5 (72–90)	48	77 (70.5–85.5)	50	78 (71–87)	0.0118*	0.360	0.3077	0.146
PR PTS (beats/min)	50	84 (72–96)	48	83.5 (76–96)	50	94 (83–103)	0.0686	0.263	≤0.0001*	0.549
SBP PS (mmHg)	50	131.5 (123–150)	48	132 (117.5–139)	50	129 (118–139)	0.0040*	0.409	0.0022*	0.425
SBP PTS (mmHg)	50	135.5 (123–148)	48	138.5 (125.5–151.5)	50	140.5 (126–151)	0.5949	0.078	0.1693	0.195
DBP PS (mmHg)	50	83.5 (77–92)	48	82 (75–87.5)	50	79.5 (73–87)	0.0438*	0.290	0.0006*	0.475
DBP PTS (mmHg)	49	82 (74–89)	47	78 (73–87)	49	77 (69–85)	0.0091*	0.376	0.0004*	0.492
DP PS mmHg/beat/min	50	11014.5 (9300–12441)	48	9662 (8881–11070)	50	9988 (9009–11200)	0.0012*	0.456	0.0190*	0.330
DP PTS mmHg/beat/min	50	11213 (9856–12920)	48	11853.5 (9983.5–13720)	50	13294 (11454–14161)	0.0668	0.265	≤0.0001*	0.596
***Respiratory***										
SO2 PS (%)	50	96 (95–98)	48	96 (95–97)	50	96 (95–97)	0.5696	0.829	0.3824	0.125
SO2 PTS (%)	50	96 (94–98)	48	96.5 (96–97)	50	97 (96–97)	0.0175*	0.341	0.0240*	0.317

*Note*. PR PS: Pulse rate pre session. PR PTS: Pulse rate post session SBP PS: Systolic blood pressure pre session. SBP PTS: Systolic blood pressure post session. DBP PS: Diastolic blood pressure pre session. DBP PTS: Diastolic blood pressure post session. DP PS: double product pre session. DP PTS: double product post session. SO2 PS: Pulse oximetry pre session. SO2 PTS: Pulse oximetry post session. P1: modifications between the start of the intervention and 12 sessions. P2: modifications between the start of the intervention and 24 sessions.

‡ : Effect size by Cohen's d.

All data was analyzed with pared Wilcoxon test.

## DISCUSSION

This telerehabilitation program in survivors of COVID-19 showed significant improvements in some of the parameters evaluated. The SMI is considered an indicator of frailty in the population over 50 years of age (Kim et al., 2018). Due to the consequences of COVID-19, a reduction in this indicator is to be expected, increasing the risk of frailty in the population (Hasegawa et al., 2021). The results of our study showed a significant increase in the SMI at 24 sessions. It is noteworthy that the SMI has been related to cardiorespiratory fitness and the prevalence of coronary artery disease in older people (He et al., 2020).

At the start of the telerehabilitation program, the median for the Barthel index was 95 (range 85-95). However, at the end of the 12 sessions, all of those evaluated had an index of 100, considered totally independent for activities of daily life. This index, originally used in the elderly population, has now been applied to surviving COVID-19 patients involved in both hospital and remote rehabilitation (Sakai et al., 2020). Further, the level of dyspnea at rest recorded during the evaluation sessions was significantly reduced at 24 sessions.

The SPPB has been widely used to determine functional status and physical performance mainly in the elderly population. However, with the prolonged stays of patients in intensive care units, its application to patients with sequelae of COVID-19 has increased (Paneroni et al., 2021). Our patients presented a significant increase at 12 and at 24 sessions, all of them reaching the maximum test value.

On the other hand, the 2MST has been shown to be an alternative for the assessment of aerobic capacity in the elderly population (Bohannon & Crouch, 2019). Our results have shown significant changes, by indicating that better aerobic capacity is related to better performance in activities of daily living (Driehuis et al., 2018). So far, no results of this test have been reported in COVID-19 survivors, so our results will be useful for future research.

Hand strength has been associated both with frailty and an increased risk of mortality. It has been suggested that hand strength could provide relevant information for the follow-up of patients with COVID-19 and signal the need for a closer surveillance of those with low levels of gripping force (Cheval et al., 2021; Ekiz et al., 2020). In our study, we observed a significant increase in hand strength.

The strength of the upper limb is related to physical independence. Higher levels of force in the elbow flexors have been related to an increase in the quality of life of patients recovering from a stroke (Lieshout et al., 2020). Our study showed an increase in 1RM for the elbow flexors. The initial increase, may reflect the low levels of elbow flexor force in these patients after suffering from COVID-19. It has been reported that some patients who have been pronated during their stay in the intensive care unit, may present alterations in the brachial plexus as a result of sustained pectoral pressure in this position, presenting as weakness in the elbow flexor muscles (Le et al., 2020).

Standing up and sitting down from a chair is one of the daily activities that represents physical independence (Bohannon et al., 2010). A ≥ 2 repetitions number increases have been reported as a minimal clinically important difference in pulmonary patients (Zanini et al., 2019). In our study 3 and 7 more (median) repetitions were performed at 12 and 24 sessions respectively. An increase in performance in standing and sitting tests has been related to an increase in gait speed in older adult women (Yanagawa et al., 2016). In addition, a higher 30-s CST performance has been associated with higher hand strength and higher endurance capacity measured through the 6-minute walk test in adults ≥ 50 years (Yee et al., 2021).

Alterations in cardiovascular and respiratory parameters at rest have been reported in survivors of COVID-19. In our study, we observed a reduction in resting pulse rate at 12 sessions, without a decrease after 24 sessions, indicating a possible stabilization of the variable following 12 sessions. Systolic and diastolic blood pressure and the double product were significantly reduced at 24 sessions, which can be associated with a reduction in resting cardiac stress (Schutte et al., 2013). On the other hand, the recording of pulse oximetry is important for patients with the disease due to the existence of cases in which a drop in oxygen saturation is not accompanied by the condition of dyspnea, which has been called “silent hypoxemia” (Zubieta-Calleja & Zubieta-DeUrioste, 2020). In our study we found no changes in pulse oximetry during the resting condition. In contrast, the post-session pulse oximetry value experienced a significant increase from the initial evaluation to session number 24 indicating that there was a decrease in the number of patients who experienced a decrease in saturation during the session. This is relevant, since it has been described in patients with COVID-19 that post-exercise desaturation is related to the severity of the disease (Goodacre et al., 2020). On the other hand, post-effort desaturation has been proposed as a variable of interest related to the severity of the presentation of lung disease (Briand et al., 2018).

Concerning the limitations of our study, information was not available on physical tests and body composition prior to the disease, so it was not possible to consider a pre-disease baseline state. We did not follow up on the dietary habits of the survivors that could explain increases in anthropometric variables.

In conclusion, a telerehabilitation programs for people who fell ill with COVID-19 allows the recovery of physical independence and increases in both SMI and physical fitness. Extending the use of telerehabilitation for this type of patient is recommended due its feasibility, safety, and low cost. The impact of telerehabilitation on the longer-term maintenance of the functionality, physical fitness, skeletal muscle mass index, and physical activity level of people after falling ill with COVID-19 remains to be assessed.
